# Allopathic Ignorance and Arrogance

**Published:** 1889-05

**Authors:** B. Fincke


### ALLOPATHIC IGNORANCE AND ARROGANCE.

The building of the first German railroad (Nuremberg-Fürth) which was opened December 7th, 1835, was sternly opposed by the highest medical authority in the land, the "Obermedicinal Kollegium in München," in a plenary assembly, which decreed that "*the running of steam-cars had to be forbidden unconditionally in the interest of the public hygiene. The rapid motion produces without fail a disease of the brain of the passengers. However, if it should not be desired to prevent those who would not hesitate to expose themselves to such a danger of getting sick, yet it remains the duty of the government to protect all those non-passengers who might look at the cars, for the mere look at a rapidly-moving train of cars would cause exactly the same disease, and, therefore, it is required at least that every railroad ought to be inclosed in a tight fence at least ten Bavarian feet high.*"

This document is in the possession of the Direction of the Nuremberg-Fürth railroad, but was not printed in Hagen's book, "Die erste deutsche Eisenbahn" (*Hom. Mon. Bl.*, Stuttgart, March, 1889, p. 44).

The *Homœop. Monatsblätter*, Stuttgurt, March, 1889, p. 45, gives the following letter:

"Treated for a complaint of the larynx (polypus of the vocal chord) without success for one and a half years, I finally went to Berlin for an operation. After examination by the most celebrated specialists, Professor Virchow stated that the disease was of a cancerous nature, and I was informed that the larynx must be cut open from outside and the diseased parts removed; if I should survive the operation, my speech, of course, would be gone forever, and the duration of my life would be in God's hands.

"In this sad and hopeless time, I wrote to Dr. Volbeding, homœopath, in Düsseldorf, and now I am so fortunate as to be cured of a terrible complaint without any operation, merely by taking the medicine of the gentleman mentioned.

"D. Zülon, Master-saddler.

"Hagenon, Meckl.-Schwer., December, 1888."

This case was published by Dr. Virchow in the *Deutsche Med. Wochenschrift*, No. 8, with the addition of a lengthy explanation, the end of which is here given literally :

"As I have learned from credible authority, the patient has not submitted to the partial extirpation of the larynx proposed at that time, but went home and has, by letter, sought the advice of the homœopath, who, in the same way, sent him advice and remedies without having seen the patient before or afterward.

"In the latter days a new examination of the patient has been made by Professor Krause himself, on account of sickness of the assistant. The Professor stated that a cure had not taken place, though the patient at present, therefore *more than fourteen months after the endo.-laryngeal tumor has no subjective complaints whatever*, except a permanent hoarseness, yet the examination with the laryngoscope proved that the disease continues unchanged in its nature.

"RUDOLF VIRCHOW.

"BERLIN, February 14th, 1886."

The disease, therefore, though not annihilated, has been so modified in consequence of the homœopathic treatment that the patient considered himself cured. More, indeed, cannot be expected in such a severe suffering and at the old age of the patient.

But Virchow and his followers do not seem to comprehend. (See *Die Münchener Nachrichten*).

ADDENDUM.

Dr. Zoeppritz, of the "Hahnemannia," has written to the gentleman above in regard to his alleged cure and received the following answer, which speaks for itself. It is literally translated :

"HONORED SIR :—Professor Krause has been here in his own interest, and has requested me to make an examination. He has here declared that I was perfectly healthy, but that I never entirely would regain my speech, since the right vocal chord had been destroyed by Dr. Friedländer (his assistant). He was pleased at my healthy appearance. In contradiction to this Professor Virchow writes quite differently ; I do not know what to say to it. * * *

"D. ZÜLON.

"HAGENON, March 7th, 1889."

The cancer is in another place than in Mr. Zülon's larynx.

B. FINCKÉ.

